# Supplementary material for: Are carbon emissions trading and green financial instruments synergistic? -Comprehensive quantitative research based on content analysis
Source: PLoS One. 2024 Mar 7;19(3):e0298601. doi: 10.1371/journal.pone.0298601 (PMC10919601; doi:10.1371/journal.pone.0298601)
Supplement: S1 Data — (ZIP) [file pone.0298601.s001.zip › Data/地级市Z_F工作报告.zip!/╜¡╦╒/╬▐╬2╩╨/└┤╫╘╣1⁄2╓┌║┼í╛┬φ┐╦╩2╛▌═°í┐_2017.docx]

各位代表：

　　现在，我代表无锡市人民政府，向市第十六届人民代表大会第一次会议报告工作，请予审议，并请市政协委员提出意见。

过去五年工作回顾

　　第十五届人民代表大会以来的五年，面对错综复杂的宏观环境和艰巨繁重的改革发展稳定任务，在中共无锡市委的正确领导下，在市人大、市政协的监督和支持下，市政府全面落实党的十八大和十八届三中、四中、五中、六中全会精神，深入贯彻习近平总书记系列重要讲话特别是视察江苏重要讲话精神，主动适应经济发展新常态，自觉践行新发展理念，紧紧依靠全市广大人民，求真务实，开拓奋进，较好地完成了“十二五”规划各项任务，并实现了“十三五”发展良好开局。

　　本届政府履职五年来，全市经济社会平稳发展，地区生产总值由6679亿元增加到9210亿元，年均增长8.4%；一般公共预算收入由615亿元增加到875亿元，年均增长7.3%；固定资产投资由3169亿元增加到4795亿元，年均增长11.7%；社会消费品零售总额由1946亿元增加到3120亿元，年均增长9.9%；城镇和农村常住居民人均可支配收入年均增长9.3%和10.1%，分别达到48628元、26158元；居民消费价格涨幅控制在省定范围内，为高水平全面建成小康社会打下了坚实基础。

　　我们确立产业强市主导战略，保持了实体经济稳定增长。在无锡处于负重爬坡的关键阶段，市委十二届九次全会作出了重振产业雄风、加快打造现代产业发展新高地的战略部署，出台促进现代产业发展的系列政策，设立200亿元现代产业发展扶持资金，推动产业强市理念逐渐深入人心，实体经济发展实现增量提质。规模以上工业总产值和增加值多年徘徊不前的情况得以改变，2016年分别突破1.5万亿元、3000亿元，达1.51万亿元和3075亿元。企业实力不断壮大，我市入围中国企业五百强、中国制造业企业五百强的企业数均居全省第一；入围中国民营企业五百强、中国服务业企业五百强的企业数均居全省第二。质量强市示范市建设深入推进，共承担制修订国内外标准363项，2家企业获全球卓越绩效奖，3家企业获中国工业大奖，荣获国家级质量奖6个、驰名商标超过80件。全市服务业增加值占地区生产总值比重由44%提高到51.3%。无锡国家数字电影产业园成为国家级文化和科技融合示范基地。五年获批国家5A级景区1家、4A级景区11家，全市旅游总收入年均增长12.2%。国家现代农业示范区建设成效明显，农业园区化比重由13.8%提高到48.9%。金融业发展取得新的进展，金融机构本外币贷款余额由7280亿元增加到10518亿元，直接融资当年发行额由181亿元增加到919亿元，境内外上市公司由74家增加到111家，新三板挂牌企业达到209家。

　　我们着力推进创新驱动发展，实现了新一轮转型升级。扎实推进苏南国家自主创新示范区建设，不断提高区域创新能力。全市拥有国家级以上工程技术研究中心6家，国家级国际合作基地10家，省级外资研发中心41家，省级国际技术转移中心8家，国家级质检中心和重点实验室21家。清华大学等高校院所在锡设立研究院21家。企业研发经费占主营业务收入比重达1.62%，全社会研发投入占地区生产总值比重由2.58%提高到2.82%，万人发明专利拥有量由7件增加到31.4件，科技进步贡献率由57.8%提高到63%，均居全省领先水平。“神威·太湖之光” 超级计算机荣获世界超算冠军，项目应用在全国首获戈登贝尔奖。扎实推进国家传感网创新示范区建设，成功举办2016世界物联网博览会，启动物联网小镇规划建设，有效推广一批物联网示范应用工程，物联网产业营业收入年均增长30%以上。高新技术产业产值占规模以上工业总产值比重由36.7%提高到43.4%。制定实施“太湖人才计划”，深入推进“人才强企”工程，成立“欧美同学会无锡报国基地”，国家“千人计划”人才、省“双创计划”人才由36人、165人分别增加到84人和407人。

　　我们坚持扩大开放不停步，优化了开放型经济发展格局。全面对接上海等自贸区试验政策，复制推广48项改革创新事项。实施外贸稳增长系列政策意见，在国际经济环境复杂严峻的形势下，保持进出口总额稳定在700亿美元左右。服务外包业务执行金额由36.5亿美元增加到102.9亿美元。加大利用外资力度，累计到位注册外资171亿美元，其中制造业外资占比达60%，引进超亿美元项目131个，中芯长电半导体、三星SDI偏光板、药明康德生命科技园等一批重大外资产业项目成功落户。支持企业“走出去”，累计对外投资74.2亿美元。柬埔寨西港特区首期建设基本完成，进驻各类企业107家。加强口岸功能建设，无锡航空口岸扩大对外籍飞机开放，进境食用水生动物、进口肉类指定口岸获国家批准，苏南硕放国际机场二期工程建成投用，国内国际客货航线由29条增加到68条。推进开发区转型升级，宜兴经济技术开发区升级为国家级开发区，无锡高新区出口加工区、江阴保税物流中心升级为国家级综合保税区。加强国际交流合作，国际友好城市由38个增加到48个，荣获国际友城“特别贡献奖”和“交流合作奖”。成功举办第四届世界佛教论坛和两届全球锡商大会，组建全球锡商联盟。

　　我们落实全面深化改革各项任务，化解了许多发展中的难题。扎实推进供给侧结构性改革，粗钢、水泥产量较近年同期最高点分别下降21.9%、10.8%，市区商品住宅去化周期大幅下降，规上工业资产负债率下降至53.4%，水电气等企业生产要素成本有效降低，“营改增”试点五年累计减税138亿元。五年来共处置化解银行业金融机构不良贷款990亿元，2016年末银行业金融机构不良贷款率为1.56%，由一度全省最高基本下降到全省平均水平。协同推进“放管服”改革，不断深化商事制度改革，累计取消下放各类行政审批项目428项，新增内资企业数和注册资金额由17669家、770亿元分别增加到37786家、2379亿元。行政区划调整和政府机构改革取得突破，梁溪区、新吴区正式成立，工商管理、质量监督、食品药品监管体制调整到位，在省内率先建成“一办三中心”政务服务体系。理顺社会事业管办分离体制和运行机制，成立市文化旅游产业发展集团、文化发展集团、体育产业发展集团。深化医药卫生体制改革，实行药品零差价销售，医联体建设实现全覆盖。深入推进金融改革，中韩（无锡）科技金融服务合作区成立，江苏民营投资控股有限公司、开鑫贷融资服务江苏有限公司落户无锡，成立江苏资产管理公司、国联人寿保险有限公司、市金融投资公司、江苏互联网金融资产交易中心，新增银行业金融机构10家、保险公司机构11家、股权投资企业59家、融资租赁公司14家、基金管理企业40余家。深化农村改革，基本完成土地承包经营权确权颁证，深入开展集体资产股份制合作改革，43个镇级产权流转交易服务中心建成运行。天然气价格、水价及其阶梯价格改革稳妥推进。

　　我们强化城乡建设管理，促进了城乡区域协调发展。制定实施城市现代化和城乡发展一体化规划，常住人口城镇化率由72.2%提高到75.8%，建成区面积由289平方公里增加到332平方公里。着力推进太湖新城、锡东新城、惠山新城、马山国际旅游岛、古运河风光带等重点区域建设，一批重点项目建成投运。推进中心城区更新改造，实施棚户区（危旧房、城中村）改造127万平方米，整治提升旧住宅区1104万平方米。市区拆除违法违章建筑130万平方米，完成115条主要道路包装出新和478个背街小巷综合治理。城市给排水设施不断完善，全市污水管网由7904.79公里增加到8724.25公里，主城区生活污水集中处理率达95%以上，供水设施覆盖率达100%，城市再生水利用率由32%提高到33%。积极推进市域重大交通基础设施建设，京沪高铁、宁杭高铁无锡段、新锡澄路惠山段、望虞河大桥建成，完成锡澄运河江阴及惠山段航道整治，无锡（江阴）港跻身亿吨大港，苏锡常南部高速公路建设启动。完善公共交通体系，地铁1、2号线建成投运，1号线工程获国家优质工程“金奖”，3号线一期、1号线南延线工程开工建设，市区公交分担率达28.2%，镇村公交覆盖率保持在100%，成为全国首批“绿色交通城市”。城市防洪排涝能力不断提高，走马塘工程建成通水。深入推进美丽乡村建设，“三星级康居乡村”和“江苏最美乡村”数量居全省前列。丁蜀镇入选第一批“中国特色小镇”。智慧城市建设持续推进，成为全国首个高标准全光网城市，在全国智慧城市发展水平评选中连续四年名列前茅。

　　我们践行绿色发展理念，改善了生态文明和环境质量。制定实施主体功能区计划，全市28.7%国土面积划为生态红线保护区。构建“四位一体”耕地保护长效机制，深化节约用地“1236”战略布局，全市基本农田稳定在165.9万亩，单位建设用地GDP产出由每平方公里4.82亿元提高到6.12亿元。制定实施水、大气污染防治行动计划，出台“1+4”水环境治理实施意见，扎实开展以太湖水、大气污染和黑臭河道为重点的环境整治工程，太湖无锡水域连续九年实现安全度夏，14个国家考核断面水质达到或优于III类比例达50%，PM2.5年均浓度下降幅度完成国家考核任务。完成无锡钢铁厂、新苏机械厂原址等地块土壤污染修复工程。推进一批重点节能减排工程，实现万元GDP能耗、水耗分别下降26.1%、25.3%，化学需氧量、氨氮、二氧化硫、氮氧化物四项主要污染物排放量分别削减21.95%、20.93%、30.55%和43.59%。建立并积极落实生态补偿机制，继续实施排污权有偿使用和交易制度，推行环境污染责任保险。强化环境司法联动，查处违法案件4752起，否决劝退各类项目886个。推进106个生活垃圾分类收集、分类处置试点，推动锡东垃圾焚烧发电厂项目顺利复工。完成8820个自然村环境整治，建成25个省级村庄规划建设示范村和560个村庄环境长效管理示范村。扎实推进“绿色无锡”建设，全市林木覆盖率和自然湿地保护率分别由25%、16.6%提高到27%、50%。荣获“中国宜居城市”“国家森林城市”“国家生态市”“全国节水型社会示范区”“全国国土资源节约集约模范市”等称号，建成全国首个生态城市群。

　　我们不断增进民生福祉，增强了人民群众获得感。每年部署并完成一批为民办实事项目，推动民生和社会事业不断发展。坚持就业优先，五年实现城镇新增就业73.25万人，新引进大学生就业19.12万人，扶持自主创业5.74万人，城镇登记失业率控制在3%以内。建立城乡一体的居民基本养老保险制度，健全城乡低保标准定期调整机制，创新深度救助机制，市区城乡低保标准提高到每人每月760元，市区居民基础养老金、企业退休人员养老金、低保家庭中的重度残疾人救助标准分别提高到每月370元、2538元和912元，各项社保覆盖率达98%以上。新增保障性住房6.2万套，享受廉租房补贴家庭达9852户，市区住房保障体系健全率超过92%。制定农贸市场布局规划，全面启动新一轮农贸市场改造和综合整治。健全平价商店常态惠民机制。完善养老服务设施，新增养老床位1.5万张，每千名户籍老人拥有养老床位数达到41.2张。推进教育事业协调发展，成为全省首个所有市（县）区全部建成省学前教育改革发展示范区的地级市、全国首批通过“义务教育发展基本均衡”认定的大中城市，公办高中实现省三星级以上优质资源全覆盖。建立江南大学无锡医学院，与省教育厅合作支持太湖学院发展，引进南京信息工程大学滨江学院。优化职业教育布局，10所学校成为国家和省职业教育改革发展示范校。居民健康水平稳步提高，医疗卫生服务体系日趋完善，新建、改扩建二级以上公立医疗机构15家、社区卫生服务中心53家，全面实施家庭医生制度。积极落实全面两孩政策。成功创建第二批国家公共文化服务体系示范区，中国大运河（无锡段）成功列入世界文化遗产。成为全国全民健身示范城市和全省首批公共体育服务体系示范区。创新社会治理模式，全面推进“政社互动”，跻身全国首批社区治理和服务创新实验区，获评全国文明城市和全国未成年人思想道德建设先进城市。深化平安无锡建设，获评全国社会治安综合治理优秀市，公众安全感和群众对政法队伍满意率居全省首位。国防动员、人民防空和后备力量建设稳步推进，军转安置、拥军优抚和军民共建等工作成绩显著，荣膺全国双拥模范城“七连冠”。全面落实安全生产责任制，安全生产事故起数和死亡人数持续“双下降”。加强食品药品监管，食品检测合格率稳定在98%以上。史志、档案、侨务、外事、对台、气象、防震减灾等工作取得新成绩，妇女、儿童、老龄、红十字、残疾人等事业有了新发展。

　　我们注重加强政府自身建设，推动了行政职能和作风转变。扎实开展党的群众路线教育实践活动、“三严三实”专题教育、“两学一做”学习教育，宣传学习江苏“时代楷模”、江苏“最美人物”事迹和无锡“工匠精神”，政风建设取得积极成效。认真落实中央八项规定精神和省、市委十项规定要求，整改“四风”突出问题取得明显成效。全面推进法治政府建设，健全重大事项决策程序，完善听证制度，提请市人大常委会审议地方性法规21部，出台政府规章34部，各级政府及部门实现法律顾问全覆盖。自觉接受人大和政协监督，认真贯彻市人民代表大会及其常委会的各项决议，定期向市人大报告工作、向市政协通报情况，认真做好代表建议和委员提案办理工作。推进财政预决算及“三公经费”决算向社会公开，“三公经费”年均减少18.6%。完成党政机关公务用车改革。加强审计监督，对公共资金、国有资产、国有资源和领导干部经济责任履行情况基本实现审计全覆盖。强化政府性债务管理，建立偿债准备金制度，政府性债务控规模、降成本、防风险的措施得到较好落实。完成“六五”普法工作，成为全省首批法治城市创建工作先进市。全面落实党风廉政建设“一岗双责”，加大反腐倡廉力度，严肃查处一批违法违纪案件。

　　过去五年，全市经济发展经历了起伏变化过程。前几年，由于多种困难和矛盾交汇，主要经济指标增速逐年回落、在全省排名居后。近年来市委审时度势，适时作出发展战略调整，确立产业强市主导战略，大力推进产业强市建设，取得显著成效。特别是刚刚过去的2016年，我们积极应对各种困难和挑战，统筹做好稳增长、调结构、促改革、惠民生、优生态、防风险各项工作，基本完成了全年各项目标任务，经济社会发展呈现更多积极变化。一是综合实力稳步提升。经济运行稳中有进、稳中向好，主要经济指标增速整体摆脱了连续多年全省排位靠后的局面，多数指标增速高于上年同期。地区生产总值增长7.5%，同比提高0.4个百分点；社会消费品零售总额增长9.6%，同比提高0.4个百分点；规模以上工业总产值、增加值和实现利润分别增长3.8%、5.8%和9.6%，同比分别提高2.0、1.4和7.6个百分点；金融机构本外币贷款余额增长10.4%，同比提高4.9个百分点。一般公共预算收入增长5.4%，居全省前列；新增境内外上市公司17家，列全省第一。二是转型升级步伐加快。物联网、新能源、服务外包等新兴产业和高技术产业增速明显高于规上工业增速，旅游、文化、金融等服务业占比提高，单位土地GDP产出、科技进步贡献率全省领先。服务业增加值占地区生产总值比重突破50%，同比提高2.2个百分点。三是人民生活不断改善。城乡居民人均可支配收入分别增长7.8%和8.3%，基本建成城乡一体的社会保障体系，社会保障水平进一步提高。脱困转化经济薄弱村30个。全面完成10件80项为民办实事项目。有效抗击夏季特大洪水灾害，社会安定有序，未发生较大以上安全生产事故。四是环境质量持续优化。组织开展新一轮太湖治理和黑臭河道整治，太湖无锡水域水质和161条重点河道水质进一步改善。空气质量优良天数比例达66.9%，同比提高2.8个百分点，PM2.5平均浓度同比下降13.1%。万元GDP能耗下降3.4%，主要污染物排放削减量全面完成省下达的任务。五是改革开放迈向深入。供给侧结构性改革、“放管服”改革、国有企业改革等取得新进展，“营改增”范围顺利扩大，通过结构性减税和降息减费等为企业降低成本200多亿元。医药卫生体制改革取得成效，医保参保群众政策范围内个人负担率下降近5个百分点。对外开放水平不断提升，外贸进出口总额698亿美元、出口总额429亿美元，分别增长2%和1.6%，居全省前列；到位注册外资34亿美元，增长6.3%。柬埔寨西港特区成为“一带一路”合作样板园区。苏锡常南部高速公路、锡东垃圾焚烧发电厂等一批事关长远的重大基础设施项目启动实施，为今后一段时期无锡经济社会发展打下了良好基础。

　　各位代表，过去五年，我们在继承中发展、在发展中创新，取得了来之不易的成绩。这主要得益于我们坚持贯彻中央和省委省政府决策部署不动摇，着力推动各项事业发展迈上新台阶；得益于市委准确定位无锡负重爬坡的阶段性特征，坚定不移推进产业强市建设，以此凝聚全市上下加快发展共识，激发干部群众攻坚克难士气，并坚持问题导向，用改革办法解决了经济社会发展中面临的许多突出矛盾和问题；得益于无锡广大干部群众和企业家传承优秀历史文化和优良工商基因，不甘落后、勇于创新、善作善成，努力探索一条适合无锡特点的转型发展之路。

　　在此，我代表无锡市人民政府，向全市人民，向辛勤工作在乡镇街道、社区、企业等基层组织和单位的广大干部员工，向人大代表、政协委员，向各民主党派、工商联、各人民团体和各界人士，向中央及省在锡单位，向驻锡人民解放军、武警官兵和公安干警，向所有参与、支持和关心无锡建设与发展的海内外朋友，表示衷心的感谢并致以崇高的敬意！

　　同时，我们也清醒地认识到，我市经济社会发展仍然存在一些困难和问题，主要是：供给结构与需求结构不相适应，经济稳定运行的基础还不牢固，中小企业生产经营困难；新增重大产业项目不多，自主创新能力不强，人才引进培育与产业强市需求仍有差距，新旧动能转换尚需加快推进；资源环境问题形势严峻，环境污染治理、节能减排和土地节约集约利用力度尚需加大；基础设施建设和城市更新改造步伐放缓，城乡区域协同发展水平有待进一步提升；居民持续增收难度增加，低收入群体和经济薄弱村脱困致富任务艰巨；一些政府工作人员服务和担当意识不强，行政审批环节多、效率低、推诿扯皮问题依然存在等。对此，我们将进一步增强问题导向和忧患意识，正视矛盾，直面问题，努力在今后工作中采取有效措施加以解决。

今后五年奋斗目标和总体要求

　　今后五年是无锡高水平全面建成小康社会的决胜阶段，是打造现代产业发展新高地、加快建设“强富美高”新无锡的关键时期。当今世界政治、经济格局正在发生广泛而深刻的变化，不确定性因素增加，全球经济复苏过程仍然曲折复杂。我国经济发展新常态特征更加明显，区域和行业走势分化，经济下行压力仍然较大。无锡经济持续增长的资源环境约束加剧，发展中遇到的问题和矛盾比其他地区更为突出。但总的看，今后五年我国仍处于可以大有作为的重要战略机遇期，无锡发展趋势也符合中央作出的这个基本判断。去年召开的省、市第十三次党代会为无锡未来五年发展指明了方向。新一届市政府必须切实把思想和行动统一到落实中央和省、市委决策部署上来，一步一个脚印把市党代会勾画的美好蓝图变成生动现实，以优异成绩向全市人民交上一份满意的答卷。

　　今后五年政府工作要全面贯彻党的十八大和十八届三中、四中、五中、六中全会精神，深入贯彻习近平总书记系列重要讲话精神，认真落实省、市第十三次党代会精神，按照“五位一体”总体布局和“四个全面”战略布局，切实树立和贯彻新发展理念，适应把握引领经济发展新常态，坚持稳中求进工作总基调，坚持以提高发展质量和效益为中心，牢牢把握深化供给侧结构性改革这一主线，牢牢把握打造现代产业发展新高地这一关键，牢牢把握保障改善民生这一宗旨，坚定实施六大发展战略，协调推进经济建设、政治建设、文化建设、社会建设、生态文明建设，聚力创新，聚焦富民，高水平全面建成小康社会，奋力谱写“强富美高”新无锡的精彩篇章。

　　今后五年的总体目标是：完成“十三五”发展各项目标任务，高水平全面建成小康社会，提前实现地区生产总值和城乡居民人均收入比2010年翻一番。到2021年，高新技术产业产值占规模以上工业总产值比重达46%，服务业增加值占地区生产总值比重达55%，全社会研发投入占地区生产总值比重达3.1%。万元GDP能耗累计下降幅度、主要污染物排放量削减幅度达到国家和省考核要求。

　　实现上述目标任务，在今后五年政府工作中，要注重把握和落实好以下总体要求：

　　一是牢牢把握稳中求进工作总基调。稳中求进工作总基调是治国理政的重要原则，也是做好经济工作的方法论，必须贯彻落实到经济社会发展各方面和全过程。要全力稳定经济运行、稳住社会大局，坚决守住不发生系统性金融风险、生态环境风险、社会风险和重特大安全事故的底线。要积极进取、奋发有为，切实做到各项工作在稳的前提下取得新的进展。

　　二是认真落实“两聚一高”新要求。围绕省第十三次党代会提出的目标任务，把发展取向、工作导向、奋斗指向更加鲜明地放到“聚力创新、聚焦富民”上。大力推动以科技创新为核心的全面创新，使创新成为无锡最鲜明的时代特征；更加自觉地践行以人民为中心的发展思想，让百姓得到更多实惠、有更强的获得感，在高水平全面建成小康社会上迈出坚实步伐。

　　三是大力实施六大发展战略。按照市第十三次党代会部署要求，坚定实施创新驱动核心战略、产业强市主导战略、全面开放战略、新型城镇化和城乡发展一体化战略、可持续发展战略、民生共建共享战略，加大推进力度，狠抓工作落实，确保落地见效，促进经济平稳健康发展和社会和谐稳定。

　　四是深入推进供给侧结构性改革和重点领域改革。继续推动“三去一降一补”五大任务落到实处，有效化解经济运行中的结构失衡、风险积累等问题。全面贯彻中央和省各项改革部署，加快推进重点领域和关键性改革，积极实施我市承担的先行先试改革任务，不断增强经济社会发展的动力和活力。

2017年主要工作

　　今年是党的十九大召开之年、供给侧结构性改革的深化之年，也是全面落实省、市党代会部署的开局之年，做好今年政府工作意义重大。按照市委十三届二次全会部署要求，今年全市经济社会发展主要预期目标是：地区生产总值增长7%—7.5%；一般公共预算收入同口径增长5%左右；固定资产投资增长5%左右；社会消费品零售总额增长10%左右；外贸进出口实现正增长；城乡居民收入增长与经济增长同步，城镇登记失业率、居民消费价格指数控制在省定范围内；节能减排和大气、水环境质量确保完成省下达的目标任务。确定上述主要预期目标，既是落实中央和省、市委决策部署以及“十三五”规划的要求，也充分考虑了宏观经济形势变化、供给侧结构性改革要求和无锡发展的阶段性特征，体现了产业强市的工作导向和发展趋向。围绕实现以上目标，今年重点做好八个方面工作：

　　（一）毫不松懈抓好产业强市建设

　　加快构建现代产业体系。围绕建设具有国际竞争力的先进制造业基地，以发展具有比较优势的战略性新兴产业为优先选项，实现新兴产业产值增长10%以上；以发展具有领先优势的智能制造为主攻方向，积极开展“中国制造2025”苏南城市群试点示范，制定实施智能制造三年行动计划，实现全市规模以上工业总产值增长3.5%、工业增加值增长6%左右；以发展具有特色优势的现代服务业为重要取向，推进省服务业综合改革试点，实现服务业增加值占比提高1个百分点。弘扬无锡“工匠精神”，推进标准、质量、品牌、信誉“四位一体”建设，新认定省级以上品牌80个，主导和参与制修订国内外标准50项以上。实施全域旅游和“旅游+”发展战略，加快万达文旅城、阳山田园文旅小镇、江阴海澜国际马术旅游区、宜兴阳羡生态度假区等重大旅游项目建设，加强文化旅游资源整合，扩大旅游产品有效供给，实现旅游总收入增长10%。因地制宜规划建设一批特色小镇和旅游风情小镇，加快特色主导产业发展，使之成为具有明确产业定位、文化内涵、旅游功能、社区特征的空间载体。

　　重视发展现代农业。加强永久基本农田保护，加快推进农业供给侧结构性改革，积极推进国家现代农业示范区和国家农业科技园区建设，稳定水稻、蔬菜种植面积，新增高标准农田1万亩、高效设施农业（渔业）1.5万亩。组织实施粮食收储供应安全保障工程。积极发展优质、高效、绿色农业，减少化肥、农药使用，加强农产品质量安全和品牌建设。加快发展“互联网+”农业，促进农业与二三产业融合发展。

　　保持有效投资稳定增长。强力推进重大产业项目招引，加强精准招商、大项目招商和全产业链招商，争取引进超10亿美元或50亿元重大产业项目5个以上。积极开展“稳心留根”和“亲商暖企”行动，加大产业政策兑现落实力度，提高政策扶持的针对性和精准度。放宽民间投资准入，鼓励企业扩大技改投入，实现工业投入增长10%左右。有效发挥政府投资项目和重大项目的引导带动作用，实施100个市级重点项目，当年完成投资超680亿元。

　　加大金融服务实体经济力度。着力疏通贷款流向实体经济渠道，实现社会融资规模1600亿元，其中新增本外币贷款900亿元以上，制造业贷款实现正增长。开展中小企业融资授信活动，发挥好各级政府应急转贷资金、风险补偿资金作用，努力缓解中小企业融资难、融资贵问题。积极发展股权投资基金和产业基金，发挥好太湖浦发母基金、国联产业母基金作用，引导和扶持新设产业基金规模200亿元以上。大力推进企业上市，支持上市公司开展并购重组，新增境内外上市公司10家、新三板挂牌公司50家以上。

　　（二）突出聚力创新的工作导向

　　以苏南国家自主创新示范区建设为引领增强科技创新驱动力。围绕建设具有全球影响力的产业科技创新中心，优化“一区三核多特”空间布局，把无锡高新区、江阴高新区和宜兴环科园作为示范区建设的主阵地，全面落实推广中关村先行先试政策，增强核心区科技创新能力和辐射带动能力。提升各类科技产业园区发展水平，充分发挥国家级特色产业基地、科技企业孵化器、大学生（留学生）创业园等主体功能，加快推进无锡国家数字电影产业园发展。强化企业科技创新主体地位，支持企业建设高水平研发机构，加大高新技术企业培育扶持力度，实施科技企业“小升高”培育计划，全市高新技术企业达到1800家以上。深入实施“太湖人才计划”和“人才强企”工程，着力引进和培养高层次人才、高技能人才和创新创业团队，深化人才发展体制机制改革，提升招才引智工作水平，优化人才发展环境，高层次人才总量达到11.7万人，引进科技创业领军人才企业50家以上。完善政产学研协同创新机制，发挥科技成果转化服务平台作用，推动一批高校和科研院所创新成果加快在锡转化，加快推进深海装备无锡研发基地建设。提升公共技术服务平台功能，着力打造国家级检验检测中心和重点实验室集群。构建军民融合科技创新体系，创建国家军民融合创新示范区，办好军民融合发展战略高端论坛。着力激发大众创业、万众创新活力，积极打造区域创客中心，新增一批省级以上创客空间。落实好科技创新优惠政策，加大知识产权保护力度，实现全社会研发投入占地区生产总值比重达2.86%，万人有效发明专利拥有量达到32件。

　　以国家传感网创新示范区建设为引领加快发展新一代信息技术产业。制定实施新一轮推进物联网技术创新和产业发展行动计划，高水平办好第二届世界物联网博览会，高水平推进鸿山物联网特色小镇建设，积极实施十大领域应用示范推广计划，完善公共服务平台，加强物联网关键技术和行业标准攻关，启动实施设基金、建基地、兴基业的物联网“三基工程”，实现物联网产业营业收入增长15%以上。大力推进云计算、大数据、软件服务、移动互联网、“互联网+”等数字经济、创新经济、分享经济和平台经济加快发展，启动建设国家超算中心产业园、太湖新城大数据产业园。加快推进工业化和信息化深度融合，积极引导传统产业运用先进信息技术改造升级。加快建设国家电子商务示范城市，促进商贸企业线上线下融合发展。

　　以全面创新为引领充分释放全社会创新创造潜能和激情。着力推进发展理念、体制机制、企业管理、商业模式等全方位、多层次、宽领域的大创新，让创新贯穿一切工作。着力打造更具影响力的新载体新平台，全力办好物联网、新能源、影视文化、工业设计、大学生创业大赛等重点博览会、交易会、展会、论坛，创特色创品牌。全面创新的本质是进一步推动思想解放，打破陈旧思维束缚，摆脱习惯路径依赖，充分尊重企业家和基层首创精神，营造良好创新生态环境，培育宽容失败、包容个性的创新文化，让全社会创造活力竞相迸发、创新源泉充分涌流。

　　（三）突出聚焦富民的发展取向

　　实现居民收入增长与经济增长同步。完善工资收入分配制度，建立职工工资正常增长机制。落实更加积极的就业政策，优化就业结构，城镇新增就业11万人，帮助重点就业困难人员实现就业8000人。着力优化创业环境，推进工商注册便利化，新注册登记企业2.8万家以上；建立市场化创业担保贷款机制，鼓励大学生等重点群体自主创业，扶持自主创业1万人，其中大学生自主创业1600人，实现带动就业5万人。拓展居民增收渠道，推进安置房上市，增加居民财产性收入来源。积极培育家庭农场、专业合作社等新型农业经营主体，提高农业生产经营效益、促进农民增收。实施全民参保计划，实现企业职工养老、医疗和失业保险分别扩面5万、5万和3万人以上。全面落实低收入群体、特殊困难群体社会救助政策，健全社会救助保障标准与物价上涨挂钩联动机制，持续推进深度救助，不断提高困难群体基本生活水平。加大对经济薄弱村精准帮扶力度。

　　改善老百姓生活环境。加快实施棚户区改造和旧住宅区整治，积极推进农村住房翻建。加强城市环境综合整治，开展建成区违法建设治理，加大背街小巷综合改造力度。改善居民住宅小区物业管理，进一步推进老小区电梯改造。加强城市绿化美化，建设一批城市小游园，新增绿化面积5000亩。加快建设15分钟社区服务圈、体育健身圈、便民生活圈。创新发展商业服务网点，加大农贸市场建设和改造力度。

　　完善公共服务体系。统筹城乡义务教育资源均衡配置，鼓励普通高中优质多样特色化发展，新建、改扩建一批幼儿园和义务教育学校。完善现代职业教育体系，推进高水平中外合作办大学取得突破，深化与江南大学、太湖学院合作共建，加快建设南京信息工程大学滨江学院。加快健康城市建设，完善药品零差价销售医疗服务机制，加强产科、儿科、精神卫生等专科建设，完善分级诊疗新格局。深入开展国家养老服务业综合改革试点，发挥居家养老基础作用，推动医养融合、智慧养老，鼓励各类社会资本进入养老领域。加强公共文化设施建设，推广公共数字文化，继续办好读书月、艺术节等活动，完善现代公共文化服务体系。深入开展全民健身运动，积极创建智慧体育城市，精心办好亚洲乒乓球锦标赛、环太湖国际公路自行车赛等国际重大赛事。

　　创新和加强社会治理。深入推进平安无锡、法治无锡建设，创新升级立体化、信息化社会治安防控体系，深化公共安全视频监控联网应用，努力创建平安中国示范区。扎实开展“七五”普法教育，加强社会信用体系建设，提高全社会法治素养、诚信意识和信用水平。创新基层治理模式，完善社区减负长效机制，打造“社区+”平台，推进“三社联动”。落实重大决策社会稳定风险评估制度，健全社会矛盾纠纷有效预防和多元化解机制，全力化解信访积案。深入推进食品安全城市创建，依法严厉打击食品药品违法行为。严格落实安全生产责任制，强化灾害监测预警和风险防范，加强公共安全监管，提升应急处置能力，切实保障人民群众生命财产安全。

　　（四）持续用力推动全面深化改革

　　切实坚持以供给侧结构性改革为主线。深入推进“去产能”，严厉打击地条钢等落后产能、非法产能，全面完成去产能任务。突出重点“去库存”，积极推进江阴、宜兴房地产去库存工作，加大市区商业办公用房去库存力度，鼓励在建和库存非住宅商品房向众创空间、养老地产、旅游地产转型。多措并举“去杠杆”，加强中小企业互保圈风险化解处置，妥善处置大中型企业债务风险，继续实现银行业金融机构不良贷款余额和不良贷款率“双下降”；规范各级政府举债行为，加大存量债务置换力度，加强地方融资平台风险管控。实实在在“降成本”，继续全面落实好国家和省降成本各项政策措施、实施好市及地区性降成本政策措施，保持政策连续性，扩大政策宣传面和受惠面，确保为企业降低成本200亿元以上。加大力度“补短板”，加快推进基础设施、民生保障、环境治理、公共服务等领域补短板工作，完善补短板项目储备和推进机制，增强经济社会发展的整体性和协调性。

　　切实坚持在重点领域改革上狠下功夫。深化“放管服”改革，积极开展相对集中行政许可权改革试点，组建行政审批局，探索实行“一枚公章管审批”；简化商事审批，推进证照整合；完善事中事后监管体系，全面推行“双随机、一公开”工作机制，继续推进综合行政执法体制改革；加快“互联网+政务服务”平台建设，整合规范公共资源交易平台。深化财税体制改革，研究完善促进区域统筹发展的财政体制，合理构建重大基础设施共建共担共享机制，落实国税地税征管体制改革。深化投融资体制改革，不断完善PPP项目扶持机制。深化国有企业改革，优化调整国有资本布局结构，加快发展混合所有制经济，积极推进国有资产证券化，完善国资国企监管机制。深化金融改革，加快发展地方金融机构，推进设立民营“锡商银行”、无锡金融租赁公司、无锡消费金融公司，推进物联网金融创新发展。稳步推进农村集体产权制度改革，总体完成农村土地承包经营权确权登记颁证，完善农村产权交易市场体系，深入推进集体经营性资产股份合作制改革。深化医药卫生、养老、教育、文化等领域改革，提高公共服务供给能力。抓好国家和省级改革试点任务落实，真正试出经验、试出成效。

　　（五）加快推进开放型经济发展

　　主动融入国家重大开放战略。积极参与“一带一路”建设，加强与“一带一路”沿线国家的投资合作、产能合作和服务外包合作，鼓励支持柬埔寨西港特区等无锡企业在境外投资的产业园区建设，促进对外投资健康发展。积极参与长江经济带、长三角城市群建设，加强与长江沿线和长三角城市的合作交流，更好地对接和服务上海，推动锡常泰联动发展。积极复制推广自贸区改革试点经验，扩大自贸区改革试点政策受益面。积极推进无锡国际邮件互换中心、药品进口口岸申报设立，提升口岸综合服务能力和开放水平。做好外事、侨务和台港澳工作，促进国际经贸合作与人文交流。办好第三届全球锡商大会。

　　集中力量做好外贸外资工作。着力优化外贸结构，积极发展外贸新业态、拓展外贸新空间，创建国家服务贸易创新发展示范区，推动外贸向优质优价、优进优出转变。落实新形势下吸引外资的政策意见，提升利用外资质量和效益，全面实施外商投资准入前国民待遇和负面清单管理模式，提高外商投资便利化和规范化水平。建设强有力招商队伍、拓展外资招商渠道，着力引进一批带动力强的行业龙头型、整机型、终端型项目，积极引进跨国公司地区总部和功能性机构，争取到位注册外资35亿美元，引进超3000万美元重大项目45个。

　　增强开发区（园区）功能作用。推进开发区体制机制创新，优化开发区空间布局和资源配置，推动开发区结合自身资源禀赋和产业基础，做强做大特色主导产业。提升开发区创新功能和承载能力，发挥无锡高新区综合保税区、江阴综合保税区载体优势，支持惠山经济开发区、江阴临港经济开发区升级为国家级开发区。优化乡镇级工业园区布局和功能。支持各级园区探索建立中外合作产业园区，推进中韩（无锡）科技金融服务合作区建设。加强开发区对标找差和考核评价工作，推动开发区综合发展水平在全省排位稳步提升。

　　（六）着力提升城乡建设管理水平

　　积极推进锡澄锡宜一体化发展。加快市域重大基础设施互联互通、共建共享步伐，开工建设苏锡常南部高速公路、南沿江高速铁路，加快推进锡澄运河市区段和锡溧漕河二期整治工程建设，积极开展盐泰锡常宜城际铁路、市域快轨S1线和宜马快速通道等前期工作，统筹搞好各地通用机场规划建设。优化市域“一体两翼”空间布局，加强产业对接和园区合作，推进锡澄锡宜文化和旅游统一规划、资源共享。支持江阴打造沿江发展带的重要节点城市，鼓励宜兴建设苏浙皖交界区域的功能性中心城市，提升澄南、宜东地区与无锡市区的融合发展水平。

　　加快基础设施建设步伐。加快实施江阴市“1310”工程。加快推进常宜、宜长高速公路建设。加密苏南硕放国际机场航线，启动货站和停机坪扩建工程，实现客货吞吐量分别突破600万人次和10万吨。争取组建地方航空公司。完善城市路网体系，加快推进蠡湖大道、江海西路快速化改造和西环线北延、新锡路北延、钱皋路工程，打通运河西路、吴桥西路、兴昌路北延等“断头路”，疏通广澄路、锡南路、鼎新路等城市道路“毛细血管”。续建地铁3号线一期和1号线南延线工程，开工建设地铁4号线一期工程，启动编制地铁第三轮建设规划。优化调整常规公交线网，推动市区公共自行车系统建设运营一体化，积极创建省公交优先示范城市。推进“海绵城市”建设，先行开展惠山工业转型集聚区等3个试点区域建设。搞好重点水利工程建设，加快建设新孟河工程和望虞河西控工程，基本完成新沟河工程，加强城市给排水和防洪排涝工程建设。发挥太湖新城、锡东新城地下综合管廊建设示范效应，加强地下空间开发利用。完善信息基础设施，推进城市大数据中心二期建设，提升智慧城市发展水平。

　　加强城市规划和历史文化保护。加快推进新一轮城市总体规划编制，促进经济社会发展规划、城乡规划、土地利用规划、生态建设规划“多规合一”。加大中心城区更新改造力度，放大行政区划调整效应，增强中心城区的商贸中心地位和历史文化保护核心区作用。加快推进太湖新城、锡东新城、惠山新城建设，做强特色产业，完善配套设施，促进产城融合。加快马山国际旅游岛、古运河风光带、鸿山省级旅游度假区建设，支持宜兴重点景区创建国家5A级景区和国家级旅游度假区。大力传承中华优秀传统文化，加强历史文化遗产保护利用，加快推进紫砂、精微绣、泥人、二胡、茶叶等历史经典产业发展，扎实推进惠山古镇祠堂群和荡口古镇申遗，提升文化传承和产业影响力。

　　提升城乡发展一体化水平。完善新型城镇化和城乡发展一体化推进机制，积极开展国家新型城镇化综合试点，加快城乡发展一体化示范特色镇建设，推动基础设施和公共服务设施向农村倾斜，促进城乡要素平等交换、合理配置和基本公共服务均等化。深入推进美丽乡村建设，开展美丽乡村休闲旅游示范村房屋改造建设试点，加强传统村落民居和历史文化名村保护，促进乡村旅游发展。全面实施居住证制度，加快土地、户籍、住房、就业、社会保障等方面制度创新，有序推进农业转移人口市民化。

　　巩固全国文明城市创建成果。健全文明城市创建长效管理机制，深入开展群众性精神文明创建活动，推动江阴、宜兴积极争创全国文明城市。大力弘扬社会主义核心价值观，发挥好道德模范、“身边好人”的示范引领作用，广泛开展争当“美德少年”等活动，加强和改进未成年人思想道德建设，打造志愿服务工作品牌，不断提高市民文明素质和城市文明程度。

（七）扎实开展“两减六治三提升”专项行动

　　落实“减煤减化”措施。减少煤炭消费总量，压减燃煤发电和热电机组数量，严格控制新建燃煤发电项目。分类整治燃煤锅炉，禁止新建燃煤供热锅炉，完成煤炭消费总量削减目标任务。大力发展清洁能源，扩大天然气利用，全面推进绿色建筑发展，推动太阳能多元化利用。减少落后化工产能，加大低端落后化工企业淘汰力度，“一企一策”实施淘汰关闭、搬迁入园、整治提升。清理并规范化工园区，推动化工企业入园进区，完成省下达的低端落后化工企业淘汰任务。

　　强化突出环境问题整治。治理太湖水环境，完善“河长制”管理，加强控源截污，狠抓农业、工业和生活污水的氮磷减排，加强清淤捞藻、应急处理和无害化处置，实现太湖无锡水域水质持续改善，确保国省考核断面水质达标。治理生活垃圾，积极推进分类收集处置体系建设，完善餐厨垃圾处理设施，推动锡东垃圾焚烧发电厂投产运营，确保生活垃圾无害化处理设施全覆盖。治理黑臭水体，完善城镇雨污分流管网设施，加强污水处理设施建设和提标改造，加大河道清淤疏浚力度，完成10条黑臭河道整治。治理畜禽养殖污染，全面整顿规范各类畜禽养殖场，实现规模化养殖场治理率达60%。治理挥发性有机物污染，突出重点园区和企业废气排放源整治，全面开展油气回收、高污染车辆淘汰和餐饮油烟污染控制工作，实现全市重点工业行业挥发性有机物排放总量较2015年削减10%以上。治理环境隐患，加强土壤污染治理与修复，加快危险废物焚烧和安全填埋设施建设，清理长江沿岸危化品码头和储罐，提升危废固废处置能力。

　　健全生态文明长效机制。提升生态保护水平，加强环太湖生态保护圈的生态修复与治理，逐步降低长江江阴段干线开发利用率，加强宜兴生态保护引领区建设，提高国家生态保护与建设示范区建设水平。提升环境经济政策调控水平，实施与污染物排放总量挂钩的财政政策，全面推行排污权有偿使用和交易，完善生态补偿机制，推进水环境区域补偿，加快实施差别化的环境价格政策；健全生态文明建设责任制，落实党政同责、一岗双责，实施领导干部自然资源资产离任审计，推行环境污染责任终身追究制度。提升环境执法监管水平，严格落实新修订的《环境保护法》，完善环境执法与刑事司法衔接机制，加大环境违法行为曝光力度，从快从严打击环境犯罪行为。

　　更大力度推进节约集约用地。落实主体功能区实施计划，严守城市边界、耕地保护和生态保护红线，严控城乡建设用地总量。不断优化土地利用结构和布局，加强产业与用地的空间协同，强化产业发展规划与土地利用规划的衔接，引导产业集聚、用地集约。着力盘活存量建设用地，加大存量土地挖潜力度，加强工业用地分类管理，推进低效用地再开发，进一步提高土地利用效率。

　　（八）努力建设人民满意的服务型政府

　　深入推进依法行政。强化法治思维，切实做到依照法定权限和法定程序行权履职。健全政府决策机制，完善重大行政决策法定程序。认真听取人大代表、政协委员意见建议，提高人大代表议案建议和政协提案的办理质量。完善审计监督机制，加强对重大政策措施落实、公共资金管理使用、经济责任履行情况的审计，依法接受市人大及其常委会的监督，主动接受市政协的民主监督，重视司法、舆论、社会监督。推进政府信息公开，动态调整并及时公布行政权力清单和责任清单，促进行政权力在阳光下运行。

　　增强担当服务意识。巩固“两学一做”学习教育成果，增强党员干部政治意识、大局意识、核心意识、看齐意识。强化责任担当，主动攻坚克难，对工作中既定的目标和作出的承诺，加强任务分解落实，确保“定了干、马上办、办到底”。改善政务服务，主动当好服务群众和企业的“店小二”，完善政府服务机制。优化“一办三中心”办事流程，切实提升服务质量和效益。深化目标任务绩效考核，建立更加有力的部门协同机制和重点工作督查机制。严肃治理“庸懒散”现象，健全容错和激励机制，营造干事创业的良好氛围。

　　切实加强廉政建设。严格执行党内政治生活若干准则、党内监督条例和问责条例，认真落实党风廉政建设“一岗双责”，深入贯彻中央八项规定和省、市委十项规定精神，驰而不息纠正“四风”，厉行勤俭节约，从严管理“三公经费”支出。更加注重源头反腐，突出公共权力运行、公共资源配置、公共资金分配等重点领域，完善内控机制，建立健全廉政建设与业务工作融合机制，加大惩治腐败力度，严肃查处发生在群众身边的腐败问题，切实解决损害群众利益的各种不正之风。

　　每年向社会承诺并组织实施一批为民办实事项目，是我们长期坚持的做法。根据今年工作安排，确定10件为民办实事项目如下：一是经济薄弱村脱困致富项目。实现全市经济薄弱村脱困转化30个以上（村级稳定性收入达到200万元）。二是黑臭水体整治项目。重点实施钱家桥河、河埒浜、许溪河、诸家桥浜、潘步桥河、铁树桥浜、唐周巷浜、民丰河、梅西河、对桥巷河等10条黑臭河道综合整治。三是城市重点道桥建设项目。开工建设新锡澄路（市区段）、兴昌路北延、通沙路北延、具区路、缘溪道、吴桥东路、中山路（青石路段）、平湖路等8条城市重点道路。四是旧住宅区综合整治改造项目。完成旧住宅区综合整治改造217万平方米。推进棚户区（危旧房、城中村）改造50万平方米。五是城市公共交通建设项目。开工建设地铁4号线一期工程。启动锡澄城际轨道S1线前期工作。加快建设城市公共自行车一期工程，投放1万辆公共自行车，建成400个配套站点。新辟和优化调整公交线路10条以上，新增、更新新能源公交车和低碳环保公交车150辆。六是农贸市场改造提升项目。新建和畅农贸市场，同步改造5个农贸市场。开展市区120家农贸市场环境整治提升工作。实现全市农贸市场食品安全快速检测网络监控全覆盖。七是健康养老服务体系建设项目。市第五人民医院建成投用。加快推进新四院建设。完成34个基层卫生医疗机构中医诊疗区（中医馆）项目的建设任务。建成投用12家街道（镇）级老年人日间照料中心。新增养老床位2000张。八是市政配套设施改造项目。完成老旧自来水管网改造60公里。完成自来水水表出户改造4000户。新增天然气用户5万户。九是文化惠民建设项目。新少年宫建成投用。建设“吴韵书香”城市阅读联盟，年内建设50个城市阅读点。完成150个多功能基层综合性文化活动室（服务中心）建设。十是教育事业基础设施建设项目。新建、改扩建幼儿园项目35个（其中新建30个，改扩建5个）、义务教育学校项目46个（其中新建24个，改扩建22个）。

　　各位代表，“两聚一高”使命光荣，产业强市责任重大。让我们紧密团结在以习近平同志为核心的党中央周围，在中共无锡市委坚强领导下，紧紧依靠和团结带领全市人民，不忘初心、务实苦干，开拓创新、继续前进，努力建设经济强、百姓富、环境美、社会文明程度高的新无锡，以优异成绩迎接党的十九大胜利召开！
